# Salt Appetite Is Reduced by a Single Experience of Drinking Hypertonic Saline in the Adult Rat

**DOI:** 10.1371/journal.pone.0104802

**Published:** 2014-08-11

**Authors:** Michael P. Greenwood, Mingkwan Greenwood, Julian F. R. Paton, David Murphy

**Affiliations:** 1 School of Clinical Sciences, University of Bristol, Bristol, England; 2 School of Physiology and Pharmacology, University of Bristol, Bristol, England; 3 Department of Physiology, University of Malaya, Kuala Lumpur, Malaysia; The University of Manchester, United Kingdom

## Abstract

Salt appetite, the primordial instinct to favorably ingest salty substances, represents a vital evolutionary important drive to successfully maintain body fluid and electrolyte homeostasis. This innate instinct was shown here in Sprague-Dawley rats by increased ingestion of isotonic saline (IS) over water in fluid intake tests. However, this appetitive stimulus was fundamentally transformed into a powerfully aversive one by increasing the salt content of drinking fluid from IS to hypertonic saline (2% w/v NaCl, HS) in intake tests. Rats ingested HS similar to IS when given no choice in one-bottle tests and previous studies have indicated that this may modify salt appetite. We thus investigated if a single 24 h experience of ingesting IS or HS, dehydration (DH) or 4% high salt food (HSD) altered salt preference. Here we show that 24 h of ingesting IS and HS solutions, but not DH or HSD, robustly transformed salt appetite in rats when tested 7 days and 35 days later. Using two-bottle tests rats previously exposed to IS preferred neither IS or water, whereas rats exposed to HS showed aversion to IS. Responses to sweet solutions (1% sucrose) were not different in two-bottle tests with water, suggesting that salt was the primary aversive taste pathway recruited in this model. Inducing thirst by subcutaneous administration of angiotensin II did not overcome this salt aversion. We hypothesised that this behavior results from altered gene expression in brain structures important in thirst and salt appetite. Thus we also report here lasting changes in mRNAs for markers of neuronal activity, peptide hormones and neuronal plasticity in supraoptic and paraventricular nuclei of the hypothalamus following rehydration after both DH and HS. These results indicate that a single experience of drinking HS is a memorable one, with long-term changes in gene expression accompanying this aversion to salty solutions.

## Introduction

The precise regulation of salt and water balance is essential for survival and good health, and when threatened, osmotic stability is aggressively defended. Integrated neuroendocrine and behavioural mechanisms function to control the excretion and consumption of water and salt in order to maintain the optimal bodily composition required for good health [1]. However, as Cordain and colleagues have pointed out [2], “the evolutionary collision of our ancient genome with the nutritional qualities of recently introduced foods may underlie many of the chronic diseases of Western civilization”. Indeed, contemporary foodstuffs are rich in salt, and habitual high salt intake is one of the unfavourable population-wide risk factors for epidemic cardiovascular disease, including hypertension [3], [4]. Interestingly, the neural organisation subserving the instinct of salt craving, which in evolutionary terms has a high survival value, appear to be plastic with respect to chronically high salt intake, resulting in enduring pathogenic changes in salt appetite and consumption [5].

The neuroendocrine reflexes regulating salt and water balance are centred on the hypothalamo-neurohypophyseal system (HNS). The HNS consists of the large peptidergic magnocellular neurones (MCNs) of the supraoptic nucleus (SON) and paraventricular nucleus (PVN), and more medial sub-division of smaller parvocellular neurons in the PVN [1]. The rise in plasma osmolality that follows high salt ingestion is detected by intrinsic MCN mechanisms [6] and by specialised osmoreceptive neurons in the circumventricular organs such as the subfornical organ, which provide excitatory inputs to shape the firing activity of MCNs for hormone secretion [7] and parvocellular neurons for activation of the sympathetic nervous system [3].

Behavioural mechanisms regulating body fluid homeostasis are determined by the ancient primal instincts of thirst and salt appetite, which are thought to be evolutionary progenitors to more complex forms of consciousness [8]. Extracellular fluid hyperosmolality, hypovolemia and the exogenous application of angiotensin II (AngII) can all stimulate the sensation of thirst, which is the powerful behavioural driver to seek and consume water [9]. In contrast, sodium loss elicits an increased motivation to find and ingest salt thus increasing salt appetite [5], [9]. To replenish body sodium animals show an exaggerated preference for palatable hypotonic to isotonic saline (IS) solutions and a willingness to ingest hypertonic saline (HS) solutions that would normally be avoided [10]. One of the primary gustatory nerves, chorda tympani (CT), plays a key role in shaping this behaviour by suppressing the salt taste response allowing increased ingestion of HS solutions [11], [12]. Accordingly, sodium deprivation or depletion in rats produces a hedonic switch in preference from water to HS solutions in choice tests [13]–[15]. Animals also drink HS solutions when offered no choice. The involuntary consumption of HS solutions has been shown to sensitise animals to drinking lower concentrations of salt in subsequent choice tests [16]. We and others have previously described transcriptional changes in the forebrain and the HNS in response to HS intake that are important in body fluid homeostasis and thus may contribute to salt appetite [17]–[20]. This capacity to ingest salty substances is vital for survival, but any lasting effects of this experience, in terms of neuronal plasticity and/or behaviour are not known.

The ingestion of high salt diets (HSD) during gestational and early post-natal phases of development increase salt intake in adult rats [21], [22]. However, maintenance on HSD after weaning has been reported not to have any long-term influences on salt intake in adult rats [23], [24]. Behavioural studies have shown that salt appetite can be enhanced, as indicated by increased HS intake, by successive rounds of sodium depletion in rats and these changes are thought to be associated with changes in neuronal plasticity in forebrain nuclei implicated in salt appetite regulation [25], [26]. It is known that eating salty foods generates a short-term shift in preference from saline solutions to water in rats [24], but this behaviour does not persist when normal food is returned in the adult [23]. The involuntary ingestion of IS or HS solutions produces a similar shift in salt preference as animals make a conscious decision to drink water as opposed to salty solutions to quench their thirst [10]. This behavioural response resembles conditioned taste aversion (CTA), though few have sought to investigate how long this behaviour persists. Taste aversion to salt solutions can be formed in adult rats by pairing the salty taste with subsequent LiCl induced sickness [27]. Such studies have demonstrated strong and persistent aversions to normally highly palatable salt solutions in adult rats [14], [27].

All told we hypothesised that acute high salt intake may alter salt appetite in adult rats. Hence, we investigated whether involuntary ingestion of saline solutions could alter salt appetite and consumption in adult rats by testing the long-term effects of a 24 h of IS and HS intake on subsequent dietary preference. We found that a single exposure to HS sensitises the animals to drinking salty but not sweet solutions in choice tests for at least 5 weeks after this drinking experience. Finally, we identified persistent changes in gene expression in the SON and PVN after 24 h of IS (or HS) intake that maybe responsible for mediating long-term changes in neuronal activity, plasticity and behaviour.

## Methods

### Animals

Male Sprague-Dawley (SD) rats were purchased from commercial suppliers (Harlan laboratories) and housed in the animal facilities at the University of Bristol. Rats with starting weights ranging between 275–325 g were maintained under a 14∶10 light dark cycle (lights on at 5 am) for at least 1 week prior to experimentation. Rats were singly housed at an ambient temperature of approximately 21°C and food and tap water were supplied ad libitum, unless stated. All salt solutions were prepared in tap water by adding the stated percentage by mass (w/v) NaCl (Melford Laboratories, UK). The HSD was custom made (International Product Supplies Limited, UK) to precisely match the formula of the standard laboratory chow (5LF5; 0.6% w/w NaCl) but contained 4% w/w NaCl. Animal experiments were performed between 0900 h and 1100 h with the exception of AngII studies that where conducted between 1600 h and 1800 h. All experiments were performed under a Home Office UK licence held under, and in strict accordance with, the provision of the UK Animals (Scientific Procedures) Act (1986); they had also been approved by the University of Bristol Ethical Review Committee.

### Experimental series 1 - Salt intake in SD rats

The ingestion of IS (0.9% NaCl) and HS (2% NaCl) solutions were evaluated in one-bottle or two-bottle tests. In one-bottle tests fluid intake was recorded for 3 d before and 7 d after the addition of salt to drinking water. For two-bottle choice tests animals were given access to two-bottles of tap water for 7 d prior to experimentation to adapt to drinking modality. On day 8, rats were simultaneously presented with one-bottle of water and one-bottle of IS, or one-bottle of water and one-bottle of HS, and fluid intake was recorded after 24 h. The preference scores, calculated by salty solution intake/total fluid intake, indicate the degree of preference for, or aversion to one-bottle compared to the other.

### Experimental series 2 – Effect of IS and HS ingestion on future two-bottle choice tests

Rats were randomly allocated into control water exposed (WEx), isotonic saline exposed (ISEx), hypertonic saline exposed (HSEx), dehydrated exposed (no fluid; DHEx) and high salt diet exposed (4% w/w salt chow plus tap water; HSDEx) groups. On day 1, drinking water was replaced with one-bottle of IS or HS for the ISEx and HSEx groups, respectively. Water was removed from the DHEx group, whereas rats in the HSDEx group were given HSD in place of standard laboratory chow for 24 h. The WEx and HSDEx group had free access to water throughout the 24 h experimental period. With the exception of the DHEx group, fluid intake was recorded for the 24 h treatment period and for the subsequent 24 h recovery period. Rats then were presented with two-bottles of water and standard laboratory chow ad libitum for 7 d. On day 9, 24 h two-bottle choice tests were performed, comparing intakes of water and IS for all experimental groups. An additional two-bottle choice test with water and IS was conducted for the WEx, ISEx and HSEx groups on day 35.

### Experimental series 3 – Salt sensitivity testing

To establish the threshold of aversion to salt solutions, a series of two-bottle choice tests were performed after initially confirming aversion to IS with descending percentage solutions 0.7%, 0.5%, 0.3%, 0.2%, 0.1%, 0.05%, 0.025%, 0.0125% and 0.00625% of salt, over the course of 19 d. Two groups of animals were used for this study, WEx and HSEx rats, with the protocol beginning on day 9 and ending on day 28 post treatment. The two-bottle choice test with water and IS was repeated on day 30 to confirm maintained aversion to IS in the HSEx group. On day 32 two-bottle choice tests were performed with water vs 1% w/v sucrose (Sigma) followed on day 34 by two-bottle choice tests with water vs 1% sucrose prepared in IS. Two-bottles of water were returned to the animals for 24 h between each choice test. All two-bottle choice tests were performed for 24 h with the positions of the bottles being alternated for each test to avoid position preference.

### Experimental series 4 – The effect of AngII on IS and water intake

The effects of AngII on IS and water intake were investigated in the HSEx group compared to WEx rats. On day 9 of the protocol from experimental series 2, rats received a single SC injection of 200 µg/kg body weight of AngII (Sigma). This dose of AngII and route of administration has previously been shown to induce thirst [25]. The animals were placed back in their home cages with one-bottle of water, one-bottle of IS and food ad libitum. All animals began drinking within minutes of AngII administration. Fluid intake was measured 1 h after AngII administration where IS was removed and replaced with water. This protocol was repeated, firstly with one-bottle containing IS, and secondly with one-bottle of water with three intervening recovery days between treatments.

### Experimental series 5 – The effects of hyperosmotic stress and rehydration on gene expression

To induce hyperosmotic stress, water was removed for 24 h or replaced by HS in drinking water for 24 h and rats were sacrificed. In a separate experiment, rats were randomly assigned into 5 experimental groups. Two groups or rats were DH for 24 h and water was returned (rehydration; RH) for 1 d or 7 d before sacrifice. Two additional groups had their water replaced with HS for 24 h and access to water for 1 d or 7 d before being killed. The control groups had access food and water ad libitum throughout the experiment. All rats were humanely killed by striking of the cranium, brains frozen on dry ice, and stored at −80°C.

### RNA extraction and cDNA synthesis

A 1 mm micropunch (Fine Scientific Tools) was used to collect SON and PVN samples from 60 micron coronal sections in a cryostat. Sections were mounted on glass slides, stained with toludine blue, and visualised on a light microscope until MCNs were evident. Using the optic chiasm (SON) or MCNs medial lateral to the third ventricle (PVN) for reference, samples were punched from frozen brain slices and dispensed into 1.5 ml tubes stored on dry ice within the cryostat. Bilateral punches were collected from 8 and 12 consecutive 60 micron sections for PVN and SON, respectively. Total RNA was extracted from micropunch samples by combining Qiazol Reagent with Qiagens RNeasy kit protocols (Qiagen). The micropunch samples were removed from dry ice and rapidly resuspended, by vortexing, in 1 ml Qiazol reagent. Following Qiazol phase separation with chloroform, 350 µl of the upper aqueous phase was removed, mixed with 70% ethanol and applied to RNeasy columns. The remaining steps were performed as recommended by the manufacturer. For cDNA synthesis 200 ng of total RNA was treated with Genomic DNA wipeout and reverse transcribed using the Quantitect reverse transcription kit (Qiagen).

### Real-time quantitative PCR analysis

The steady-state RNA levels in the SON and PVN were assessed by qPCR. All of the genes chosen for analysis have previously been shown to be regulated by high salt intake. Primers for c-Fos (5′-AGCATGGGCTCCCCTGTCA-3′ and 5′-GAGACCAGAGTGGGCTGCA-3′), orphan nuclear receptor Nr4a1 (5′-CTGCGACTGGGTCCTGGGTC-3′ and 5′-TGTCAGGTGGTCACGCGGTC-3′), Activity-regulated cytoskeleton-associated protein (Arc) (5′-GGCCGAAGGAACCTCTACTT-3′ and 5′-CTGAGAGGGGAGCTATGCTG-3′), heteronuclear arginine vasopressin (hnAVP) (5′-GAGGCAAGAGGGCCACATC-3′ and 5′-CTCTCCTAGCCCATGACCCTT-3′), mature AVP (5′-TGCCTGCTACTTCCAGAACTGC-3′ and 5′-AGGGGAGACACTGTCTCAGCTC-3′), heteronuclear oxytocin (hnOT) (5′-TGAGCAGGAGGGGGCCTAGC-3′ and 5′-TGCAAGAGAAATGGGTCAGTGGC-3′), mature OT (5′-TGCCCCAGTCTTGCTTGCT-3′ and 5′-TCCAGGTCTAGCGCAGCCC-3′), heteronuclear corticotropin releasing hormone (hnCRH) (5′-GGGCGAATAGCTTAAACCTG-3′ and 5′-CAGGTGACCCTTCCTTGGAGA-3′), mature CRH (5′-CTCTCTGGATCTCACCTTCCAC-3′ and 5′-CTAAATGCAGAATCGTTTTGGC-3′), and ribosomal protein RPL19 (5′-GCGTCTGCAGCCATGAGTA-3′ and 5′-TGGCATTGGCGATTTCGTTG-3′) were synthesised by Eurofins MWG Operon. The optimisation and validation of primers was performed using standard ABI protocols. The qPCRs were carried out in duplicate in 25 µl reaction volumes using 12.5 µl 2 Χ SYBR green master mix buffer (Roche), optimum concentrations of primers, and first strand cDNA template. The reactions were performed using an ABI 7500 Sequence Detection System (ABI, Warrington, UK), with universal cycling conditions. For relative quantification of gene expression the 2^−ΔΔCT^ method was employed [28]. The internal control gene used for these analyses was the housekeeping gene RPL19.

### Statistical analysis

Statistical differences between two experimental groups were evaluated using independent sample unpaired Student’s t tests. One way ANOVA and Tukey’s post-hoc test were used to determine the differences between more than two groups with only a single influencing factor. For 7 d one-bottle IS and HS studies, a repeated measures ANOVA with Bonferonni post-hoc test has been used. P<0.05 was considered significant. Results are expressed as mean +SEM of the number of replicates indicated in the figure legend.

## Results

### Salt intake in SD rats

**Figure 1:**
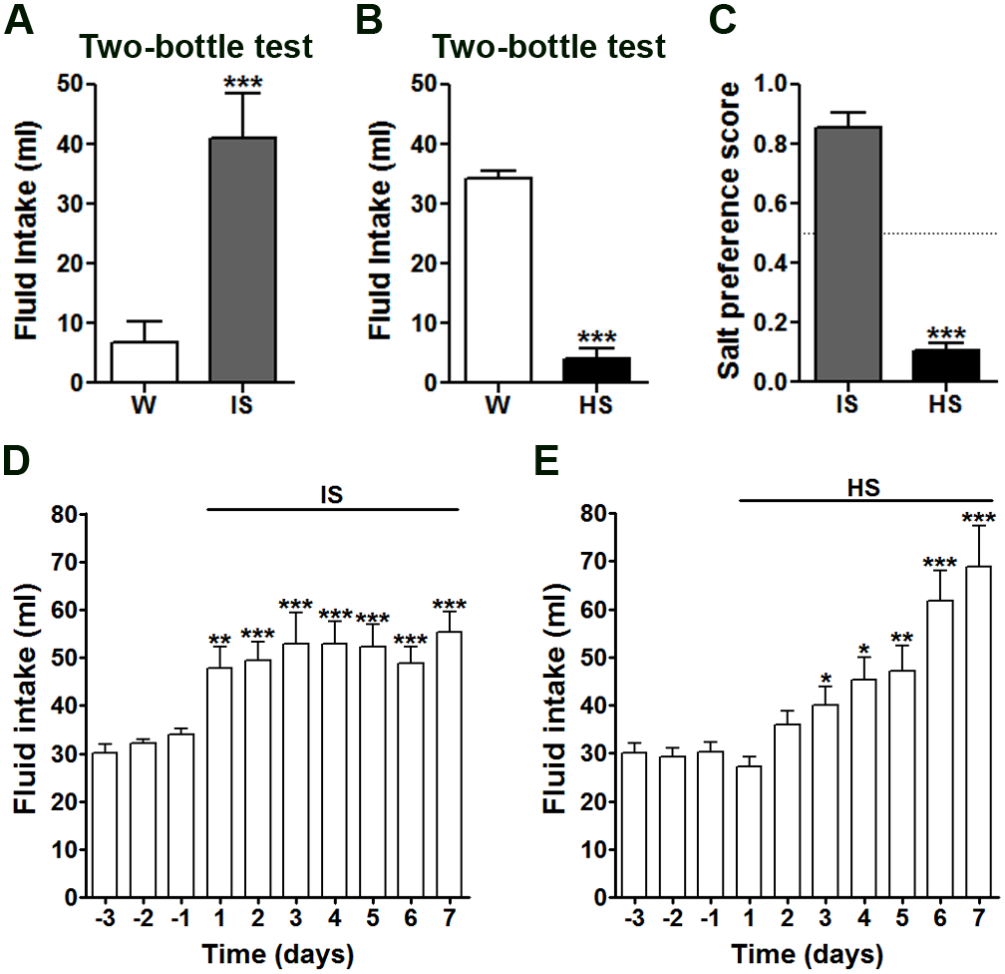
Mean fluid intake in two-bottle and one-bottle salt intake tests in naïve rats. Intakes of IS (A) and HS (B) compared to water in 24 h two-bottle choice tests in naïve rats. C, mean preference scores for IS and HS solutions in 24 h two-bottle salt choice tests with water. One-bottle intake tests with IS (D) and HS (E) in naïve rats. Error bars +SEM, n = 6–10 animals per group. *p<0.05, **p<0.01, ***p<0.001. HS, hypertonic saline; IS, isotonic saline; W, water.

In two-bottle choice tests, rats showed a strong preference for IS, ingesting significantly more compared with water (Fig. 1A). Offering HS in place of IS reversed this effect, with rats preferring to drink water (Fig. 1B). As expected, the preference score for salt significantly decreased in rats presented with HS compared to IS solutions in two-bottle choice tests (Fig. 1C). When fluid intake tests were performed with only one-bottle of IS, rats immediately (24 h) ingested significantly greater volumes of this salty solution compared with earlier water intake measures (Fig. 1D). This significant increase in fluid intake was maintained for the 7 d IS drinking test. The strong aversion for HS, as observed in two-bottle choice tests, was overcome in a one-bottle HS test (Fig. 1E). The mean intake of HS was comparable to that of earlier water intake on day 1 and 2, from which HS intake steadily increased from 3–7 d.

### Long-term changes in salt preference after ingestion of IS and HS solutions

**Figure 2:**
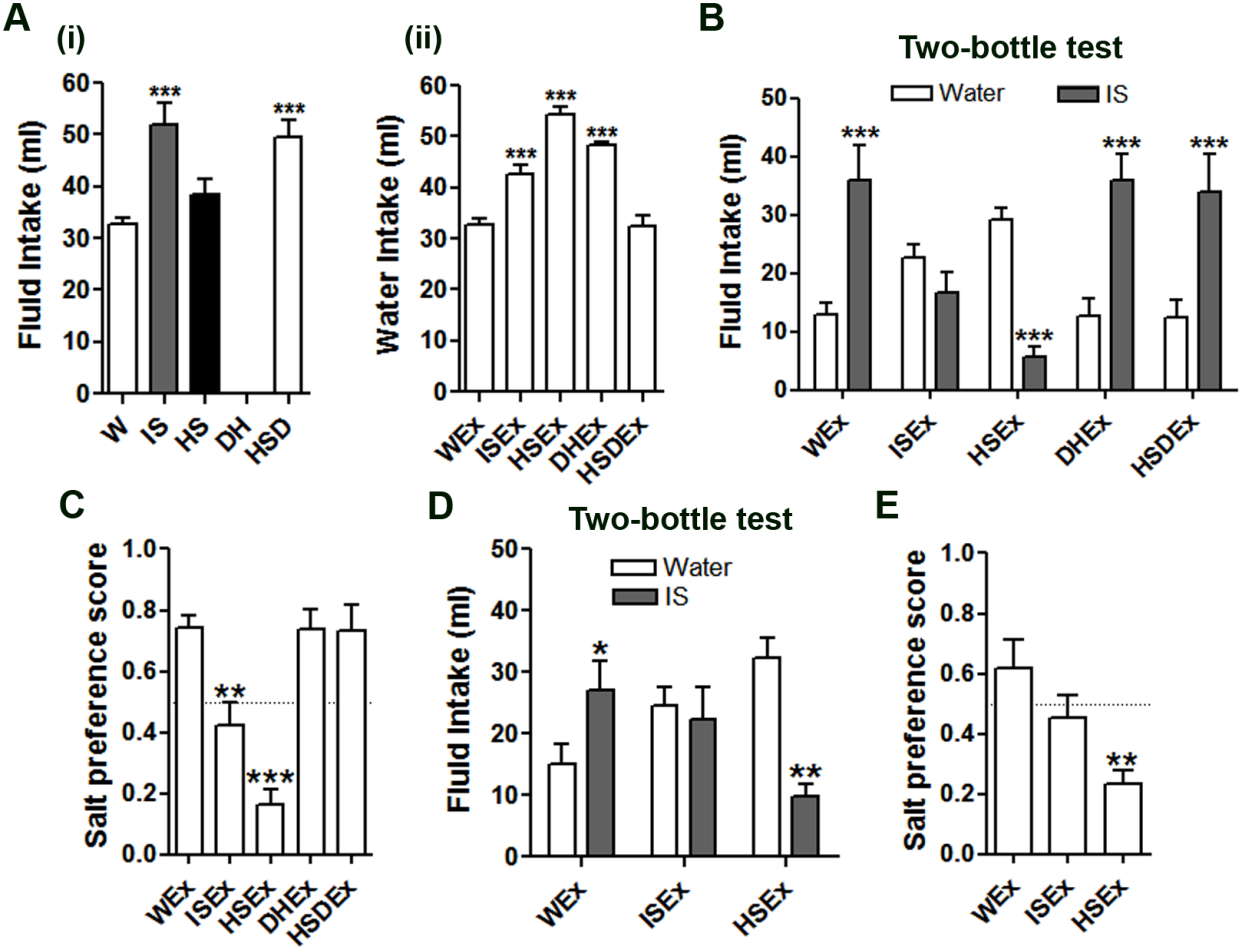
A dramatic and lasting switch in salt preference after 24 h one-bottle tests with IS and HS solutions. Ai, Mean fluid intake following 5 different 24 h one-bottle treatments, control, IS, HS, DH and 4% HSD. Aii, water intake measures for the proceeding 24 h after treatment. B, mean intakes of water and IS solution in two-bottle choice tests performed 7 d after one-bottle tests. C, mean salt preference scores for all groups calculated from the total fluid intake data in B. D, the two-bottle choice tests were repeated 35 d after one-bottle tests for control WEx, ISEx and HSEx groups. Values are means +SEM of n = 6–10 animals per group. E, mean salt preference scores for all groups calculated from the total fluid intake data in D. *p<0.05, **p<0.01, ***p<0.001. DH, dehydrated; HS, hypertonic saline; HSD, high salt diet; HSEx, hypertonic saline exposed; IS, isotonic saline ISEx isotonic saline exposed; W, water; WEx, water exposed.

Fluid intake was first measured over a 24 h period in control WEx, ISEx, HSEx, DHx and HSDx groups (Fig. 2Ai). Compared to WEx rats, fluid intake was significantly higher in the ISEx and HSDEx animals. Water intake during the 24 h recovery period was significantly higher for all experimental groups (Fig. 2Aii), with the exception of the HSDEx group. The effects of these treatments on salt appetite were then investigated 7 d later using two-bottle IS vs. water choice tests (Fig. 2B). As before (Fig. 1A), WEx animals showed a strong preference for IS compared to water. There were no significant differences in IS preference in DHEx or HSDEx treated rats compared to WEx controls. Interestingly, prior exposure to 24 h ingestion of either IS or HS solutions in the ISEx and HSEx groups respectively resulted in significant alterations in drinking behaviour. The ISEx group displayed no preference for either water or IS. In contrast, the HSEx group showed a strong preference for water over IS. Both the ISEx and HSEx rats exhibited significantly lower salt preference scores compared to control WEx rats (Fig. 2C). After a further 28 d, the two-bottle test was repeated on these same animals (Fig. 2D). We were surprised to see that, 35 days after exposure, the ISEx and HSEx groups still showed no preference for the normally very palatable IS, rather, as at 7 d, the ISEx group showed no preference, whereas the HSEx group preferred water. However, only HSEx rats exhibited significantly lower salt preference scores compared to control WEx rats (Fig. 2E).

### A loss in preference for salty by not sweet solutions after ingestion of HS

**Figure 3:**
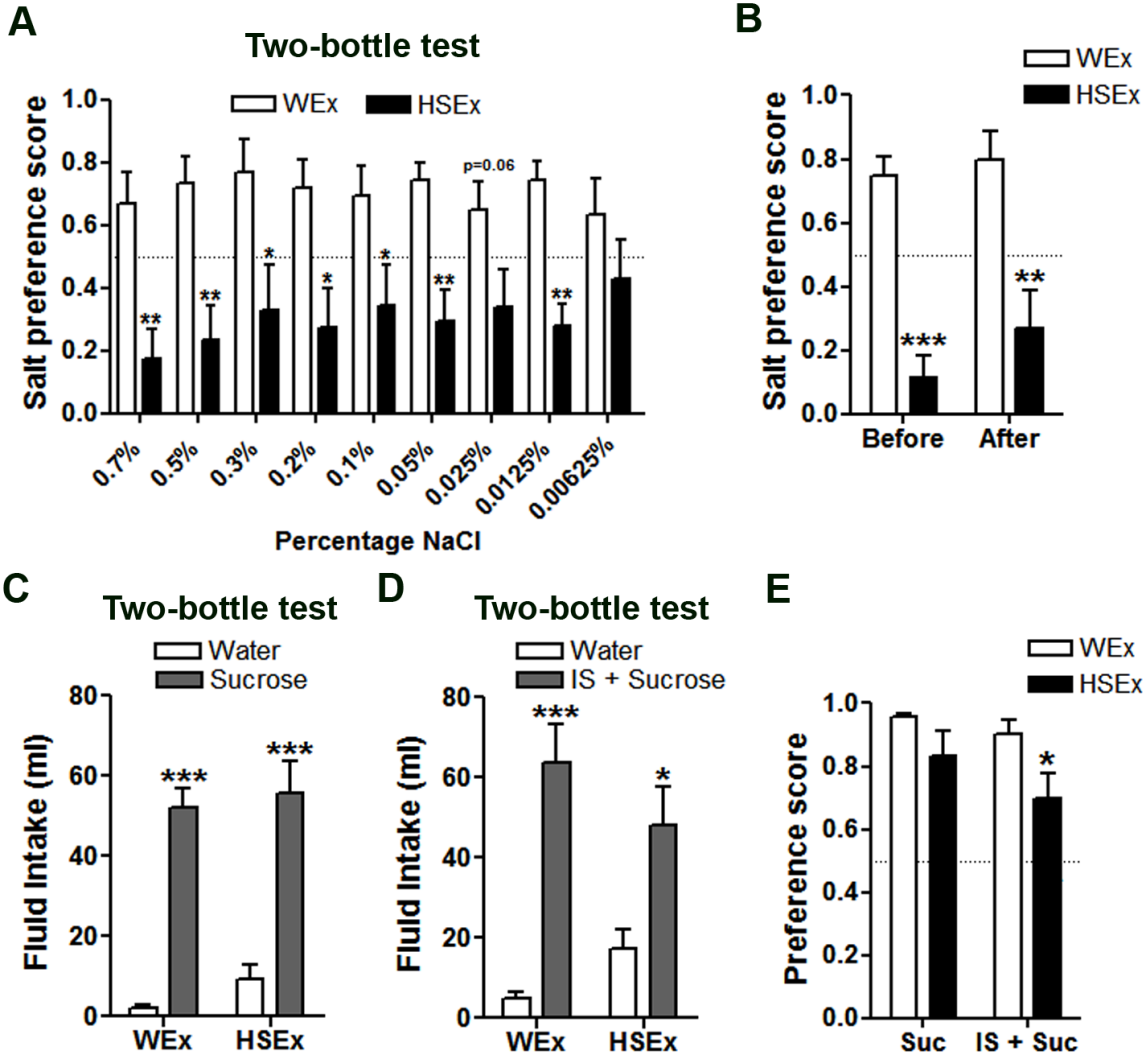
A significantly reduced appetite for salt solutions following a single 24 h one-bottle test with HS. A, Mean preference scores have been calculated from 24 h measures of water and salt intake at the percentage shown for WEx and HSEx rats. B, mean preference score for IS on day 9 (before tests) and day 30 (after tests). C, mean intakes of 1% sucrose solution compared to water in 24 h two-bottle choice test. D, mean intakes of 1% sucrose prepared in IS in 24 h two-bottle choice test with water. E, mean preference scores calculated from the fluid intake data in C and D. Values are means +SEM of n = 8 animals per group. *p<0.05, **p<0.01, ***p<0.001. HS, hypertonic saline. HSEx, hypertonic saline exposed; IS, isotonic saline; Suc, sucrose; WEx, water exposed.

To determine the threshold of aversion to salt elicited by HS, rats were presented with a descending series of salt solutions (0.7%, 0.5%, 0.3%, 0.2%, 0.1%, 0.05%, 0.025%, 0.0125% and 0.00625%) in 24 h two-bottle choice tests with water (Fig. 3A). The control WEx rats preferred the salty solution at all salt percentages tested, as indicated by mean scores >0.5. The opposite behaviour was observed for the HSEx group, which preferred water over salt solutions, as indicated by preference scores <0.5. The paired comparisons of preference scores in HSEx and WEx rats showed that HSEx rats maintained a significantly lower preference for salt solutions as low as 0.0125%. A strong aversion to IS was still evident after 30 d in HSEx compared to WEx rats (Fig. 3B). Both the WEx and HSEx rats preferred 1% sucrose solution in the absence (Fig. 3C) or presence (Fig. 3D) of IS compared to water consumption. Interestingly, the preference score significantly dropped in HSEx compared to WEx rats after addition of salt to the sucrose solution (Fig. 3E).

### The effect of AngII on IS and water intake

**Figure 4:**
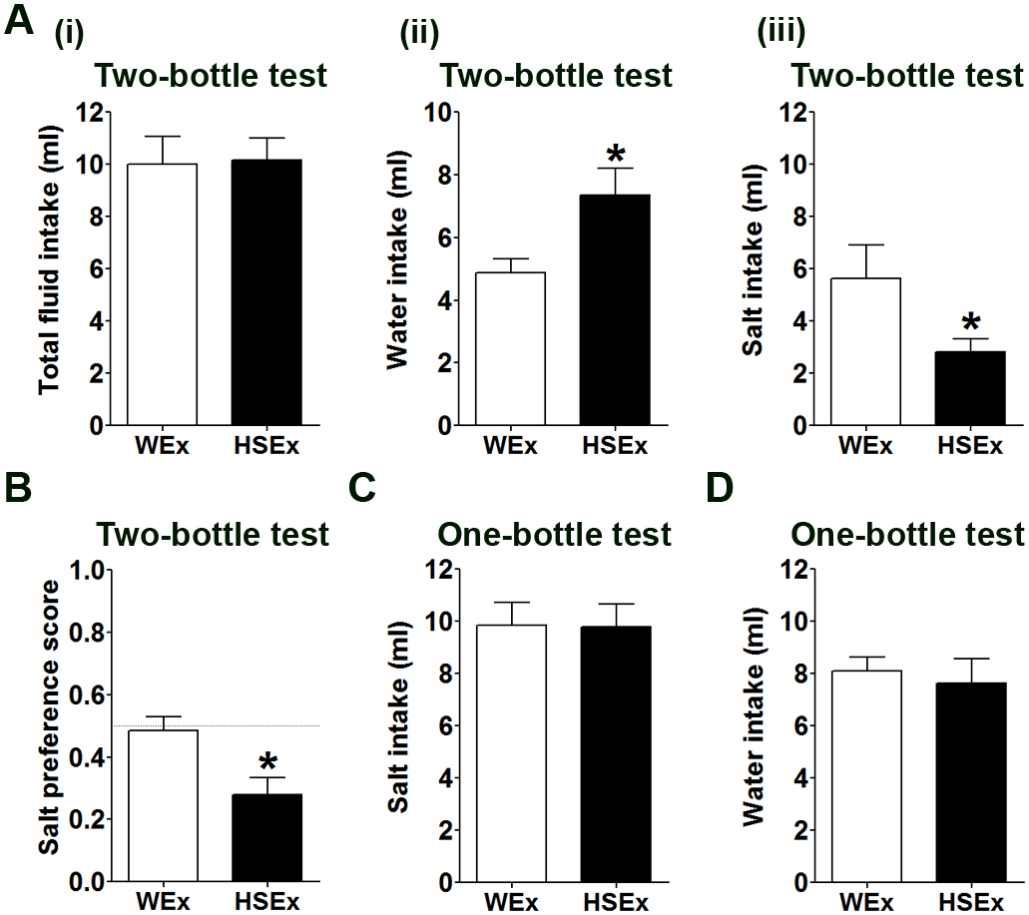
The effect of AngII on IS and water intake. Ai, total fluid intakes in WEx and HSEx rats in 1 h two-bottle choice tests with water and IS after AngII administration. Water intake (Aii) and IS intake (Aiii) in WEx and HSEx rats after AngII administration. B, salt preference score in WEx and HSEx rats after AngII administration. One-bottle tests with IS (C) and water (D) following AngII administration. Values are means +SEM of n = 10 animals per group. *p<0.05. HSEx, hypertonic saline exposed; IS, isotonic saline; WEx, water exposed.

To test whether the salt aversion in the HSEx group could be overcome by an agent known to provoke a profound thirst and salt appetite, a 1 h two-bottle choice test was performed after SC injection of AngII in WEx and HSEx rats (Fig. 4). As expected, AngII robustly increased total fluid intake in both groups (Fig. 4Ai). However, the HSEx group ingested significantly more water (Fig. 4Aii) and significantly less IS (Fig. 4Aiii) compared to WEx controls, resulting in a lower salt preference score (Fig. 4B). In one-bottle tests, no differences in water (Fig. 4C) or IS (Fig. 4D) intake were observed in when WEx or HSEx rats were given AngII.

### Profiling the effects of hyperosmotic stress and rehydration on gene expression in the SON and PVN

**Figure 5:**
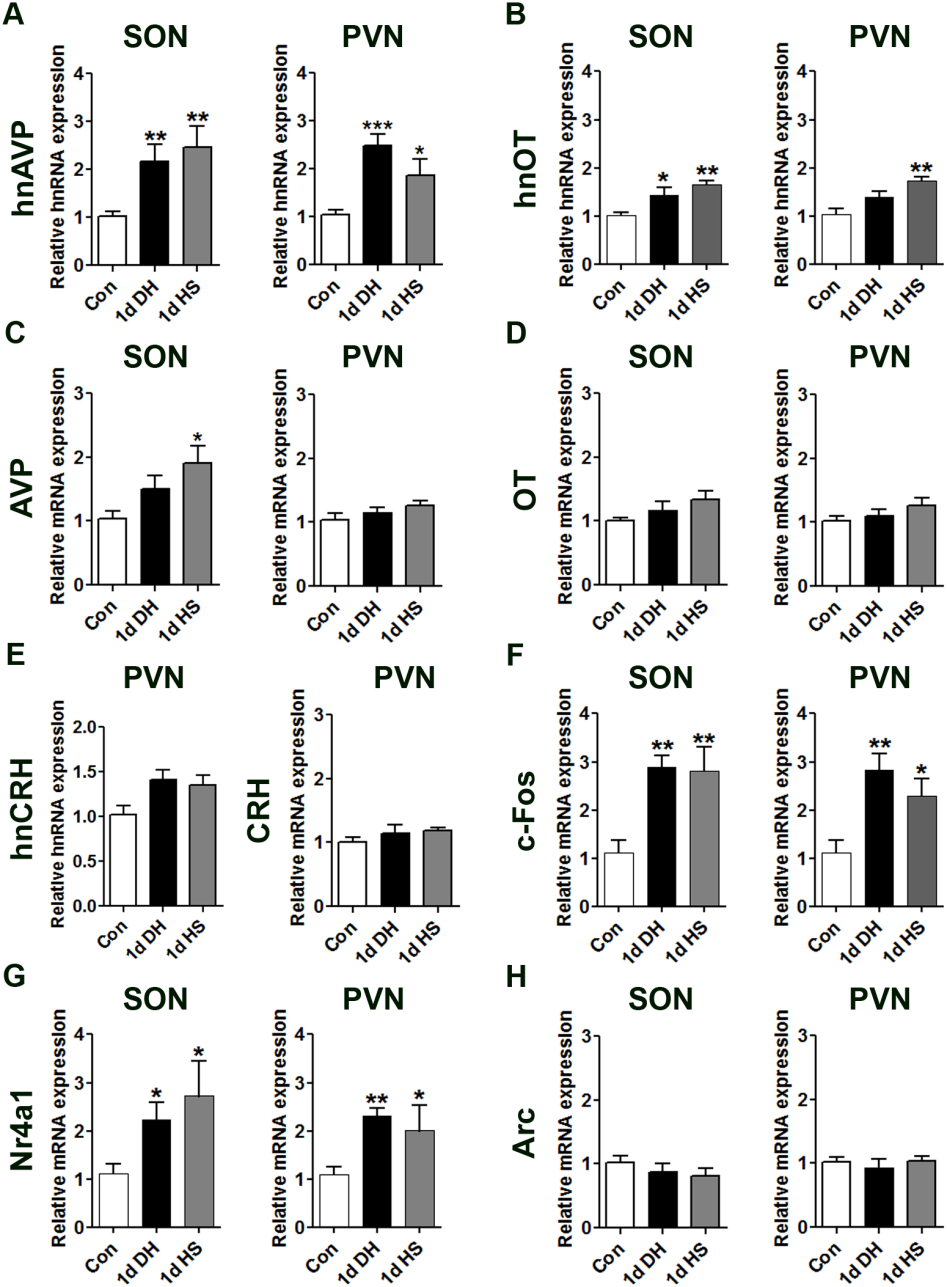
The effects of 1 d DH and 1 d HS exposure on gene expression in rat SON and PVN. Relative mRNA expression of hnAVP, hnOT, AVP, OT, hnCRH, CRH, c-Fos, Nr4a1 and Arc was investigated by qPCR in the SON and PVN of control, 1 d DH and 1 d HS rats. Values are means +SEM of n = 5–6 animals per group. *p<0.05, **p<0.01, ***p<0.001. 1 d DH, 1 day dehydrated; 1 d HS, 1 day hypertonic saline.

We used qPCR to ask if different salt ingestion paradigms altered gene expression in the SON and PVN. First we showed that both 1 d DH and HS induced the expression of hnAVP (Fig. 5A) and hnOT (Fig. 5B) in both the SON and the PVN (except 1 d DH hnOT). In contrast, mature AVP and OT mRNA expression was similar to control animals with only AVP mRNA expression being significantly increased in SONs of 1 d HS rats (Fig. 5C, D). These two paradigms did not significantly alter hnCRH or mature CRH transcripts in the PVN compared to control animals (Fig. 5E). We observed higher c-Fos (Fig. 5F) and Nr4a1 (Fig. 5G) mRNA levels in the SON and PVN of DH and HS rats, but no change in Arc (Fig. 5H) mRNA expression, compared with control animals.

**Figure 6:**
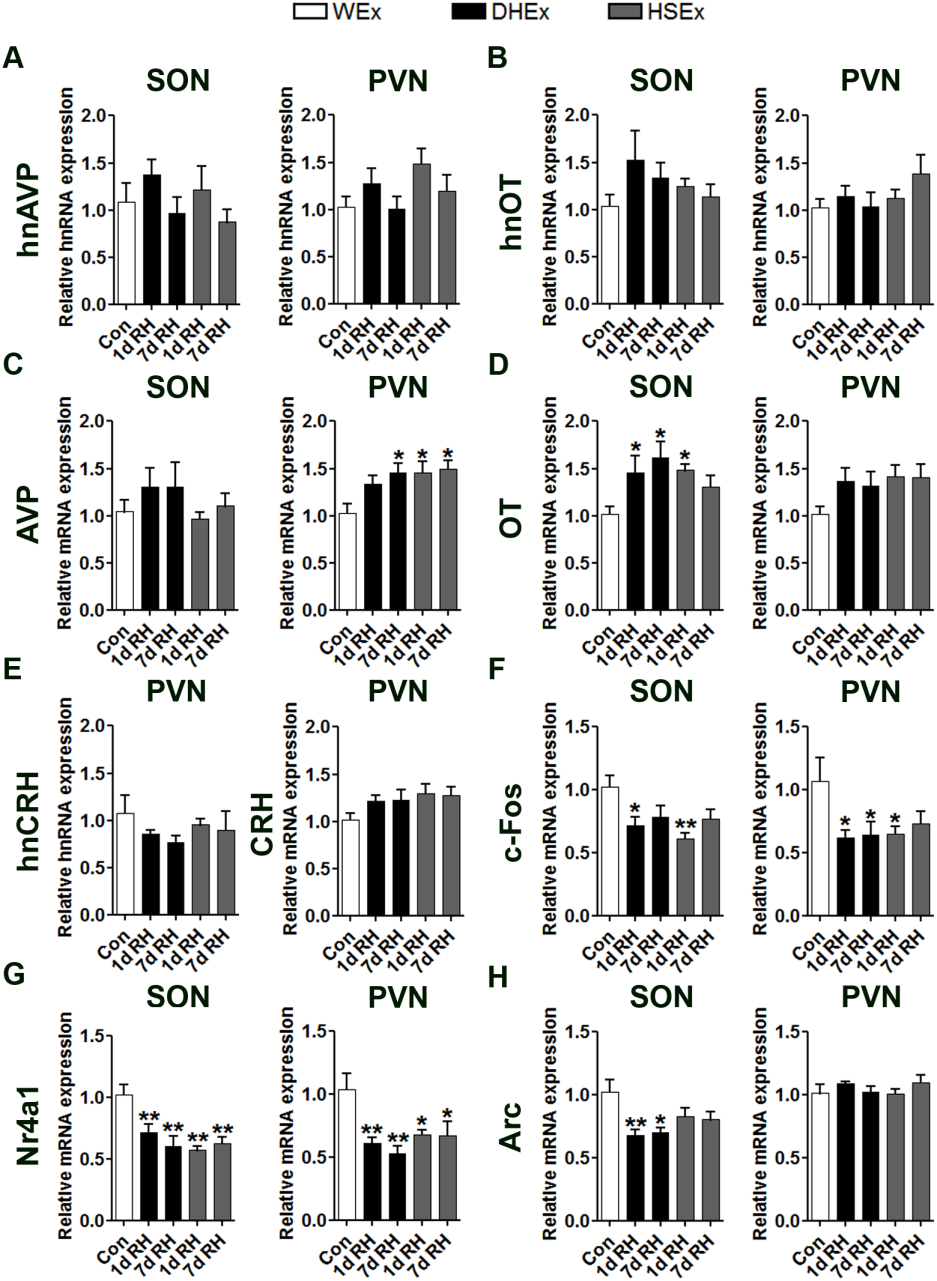
Gene expression changes in rat SON and PVN 1 d and 7 d after DHEx or HSEx compared to control animals. Relative mRNA expression of hnAVP, hnOT, AVP, OT, hnCRH, CRH, c-Fos, Nr4a1 and Arc was investigated by qPCR in the SON and PVN of rats RH (1 d and 7 d) after DHEx and HSEx. Values are means +SEM of n = 6 animals per group. *p<0.05, **p<0.01, ***p<0.001. DH, dehydrated; DHEx dehydrated exposed; HS, hypertonic saline; HSEx, hypertonic saline exposed; RH, Rehydration.

In a separate experiment, we analysed the expression of the same transcripts after 1 d and 7 d RH in 1 d DHEx or 1 d HSEx compared to control (euhydrated) animals (Fig. 6). There were no significant differences in hnAVP (Fig. 6A) or hnOT (Fig. 6B) RNA expression in either the SON or PVN following either 1 d or 7 d RH. However, the expression of mature AVP transcripts were significantly higher in PVN, but not SON, of 7 d RH following DHEx and 1 d and 7 d RH following HSEx compared to control animals, suggesting long-term alterations in the expression of this gene (Fig. 6C). OT mRNA expression was significantly higher in SON, but not PVN, of 1 d and 7 d RH following DHEx and 1 d following HSEx compared to control again supporting long-term changes in gene expression (Fig. 6D). As in 1 d DH and 1 d HS animals, no significant differences in hnCRH or mature CRH transcripts were detected in the PVN after 1 d or 7 d RH (Fig. 6E). In contrast, c-Fos mRNA expression was significantly lower in SON and PVN of DHEx and HSEx animals after 1 d RH (Fig. 6F) and in the PVN of 7 d DHEx animals. Nr4a1 mRNA levels were also significantly lower than controls at both RH time-points in both the SON and PVN (Fig. 6G). Interestingly, Arc mRNA expression was significantly lower at both time-points in SON but only for DHEx and not HSEx animals compared to control, with no significant changes in PVN (Fig. 6H).

## Discussion

In this study, we have shown that 24 h drinking of IS or HS solutions is sufficient to produce long-term changes in salt appetite in SD rats. The two-bottle choice test has been widely used to investigate the drinking behaviour of rodents in a range of experimental models [26], [29]–[31]. We performed two-bottle choice tests in naïve rats to confirm previous reports of a preference for IS over water, and an aversive response to HS (Fig. 1A–C) [15]. However, when denied a choice (involuntary drinking), naïve rats consumed large volumes of both IS and HS (Fig. 1D, E). The increase in HS intake likely results from a suppression of the taste response to HS, overriding the normal aversive behaviour, making salty solutions more palatable [13], [32]. This capacity to drink HS in an involuntary situation is vital for survival.

Although many studies have looked at the effects of sodium deprivation on salt taste responses [11], [13], [14], few have sought to address the impact of acute sodium overload on chronic salt taste sensitivity [16], [24]. Rats prefer to drink water over salt solutions when dietary sodium intake (fluid or food) is high, or immediately after HS or DH [10], but to our knowledge no lasting effects of such stressors on salt taste sensitivity have been reported. We know from behavioural experiments that multiple rounds of sodium depletion enhance HS ingestion in acute and chronic tests, suggesting that such experiences are remembered [25], [26]. Thus we asked, does prior ingestion of fluid high in salt (or food) alter the conscious decision of rats to actively seek out salty solutions in later choice tests? Accordingly, naïve rats were primed for 1 d by involuntary exposure to IS, HS, DH or HSD. Performing two-bottle choice tests 7 d post-treatment enabled rats to re-establish fluid and electrolyte homeostasis. While no changes in drinking behaviour were observed in DHEx or HSDEx groups, ISEx and HSEx groups showed significantly lower preferences for IS (Fig. 2B). This salt aversion is incredibly sensitive, with animals showing a strong preference for water over salt solutions as low as 0.0125% (Fig. 3A). Remarkably, the drinking of HS is such a memorable experience that animals continued to avoid normally palatable solutions of salt for the 35 d experimental period (Fig. 2D, E). It is not known how long this aversive behaviour to IS persists after drinking HS, or indeed if it is reversible. Further, salt aversion cannot be overcome by AngII administration (Fig. 4).

We were surprised that behavioural differences were confined to groups that ingested salty solutions. The observed changes in drinking behaviour may underlie distinct differences in mechanism provoking thirst in DH and HSD exposed compared to salty solution exposed animals. Unlike other experimental groups, rats exposed to HSD had access to water throughout the exposure period, so could immediately correct body electrolytes by altering water intake, as observed by increased water intake. In the absence of drinking fluid (DH), extracellular and intracellular fluid volumes decrease as water and sodium are inevitably lost in sweat and urine, leading to hypovolemia [10], [33]. In contrast, intake of HS solution increases body sodium content, causing an increase in the extracellular and a decrease in intracellular fluid volumes [33]. Therefore, unlike rats exposed to salty solutions [16], DH animals also display enhanced salt appetite, which occurs shortly after the initial phase of rehydration to replace body sodium [10]. No such enhancement in salt appetite is seen immediately after IS or HS exposure [16], clearly highlighting behaviour differences between these experimental paradigms in relation to salt appetite.

Although such salt avoidance characteristics are observed in Fischer 344 rats [12], [34], we were surprised that SD rats displayed aversive behaviours to a range of salt concentrations, which are normally very palatable for this strain [35]. We propose that simply drinking HS elicits a conditioned taste aversion (CTA) to IS and hypotonic saline solutions. The generation of CTA involves ingestion of a novel substance (conditioning), in our case HS, followed by malaise (unconditioned stimulus) so the animal remembers the taste of the ingested substance [27]. This is commonly achieved by administering LiCl shortly after the conditioned stimulus to generate LiCl induced sickness [27]. In agreement with our findings, these protocols produce a shift in preference for water that extends to salt solutions as low as 0.003 M [14], [30]. We can only speculate that the ingestion of HS may produce gastrointestinal discomfort leading to formation of the unconditioned stimulus. One argument against this hypothesis comes from HS intake in rats with open or closed gastric fistulas [36]. In this study, Davis et al. found that post-ingestional stimulation is the principal component in the control of water and IS intake, whereas the orosensory response appears to be controlling intake of HS. Thus, taste could be the important factor governing decision subsequent to HS consumption.

Previous studies have also shown that temperature [37] and indeed taste of fluid [38] can lead to CTA. To acquire a strong CTA to salt, the taste information is conveyed via one of the primary gustatory nerves, the CT, which is highly sensitive to salt, with increased firing rate as salt concentrations increase [39]. A decrease in CT nerve firing in sodium-deprived rats is thought to be a necessary response to increase salt intake [11], [40]. Transection of the CT in rats, as well as other mammals, diminishes salt avoidance, supporting the importance of this nerve in increasing salt appetite [12], [41]. HS drinking for 1 d will undoubtedly have increased the firing rate of the CT. However, once established, CTA to salt no longer requires the CT, as aversive behaviour to salt solutions are retained following nerve transaction [12], suggesting long-term changes in the brain.

We reasoned that these modifications to salt appetite may be attributed to changes in neuronal plasticity, similar to that described following sodium depletion [25], [26]. Thus, we asked whether gene expression is altered in the hypothalamus as a consequence of HSEx. We and others have reported changes in gene expression in the SON and PVN following hypo- and hyperosmotic stress [42]–[44]. Indeed, as expected 1 d DH and 1 d HS significantly increased mRNA expression of immediate early genes c-Fos and Nr4a1 and hnRNA for neurohypophysial hormones AVP and OT (with exception of 1 d DH) in the SON and PVN (Fig. 5), consistent with previous studies [45], [46].

Access to water after DHEx and HSEx has been shown to rapidly reduce Fos and Nr4a1 protein staining in the MCNs of the SON and PVN [45], [47]. At the RNA level, we observed significantly lower c-Fos and Nr4a1 mRNAs, by comparison with euhydrated naïve controls following RH, particularly after 7 d (Fig. 6). The higher mRNA expression of AVP and OT transcripts during RH (Fig. 6) are supported by previously reported increases in these transcripts following recovery from DHEx and HSEx [17], [48]. This is intriguing as both of AVP and OT peptides have been shown to be important in the regulation of salt appetite [49], [50]. Interestingly, another immediate early gene, Arc, was similarly down regulated in the SON during RH. This gene has been shown to function as a master regulator of protein-dependent synaptic and thus, neuronal plasticity [51]. In agreement, microarray analysis of hypothalamic genes regulated by sodium deficiency identified Arc as being significantly upregulated, implying that Arc regulation may be important for increasing salt appetite [52]. This lends support to the concept that lower Arc expression in the present study may lead to reduced salt appetite.

The changes in gene expression observed in the SON and PVN would imply that mRNAs may also be altered in additional brain centres important for salt appetite regulation. Likely future targets include the circumventricular organs such as the subfornical organ (SFO) and organum vasculosum of the lamina terminalis (OVLT). Of particular interest is the SFO, which provides excitatory inputs to shape the firing activity of MCNs for hormone secretion [7]. Being directly exposed to the chemical environment of the circulation, the SFO is able to sense sodium concentration in the body fluid through specialised osmoreceptive neurons, and is thus thought to be the principle site of regulation of salt intake behaviour [53]. In agreement, damage to SFO results in an attenuation of induced salt appetite by AngII [54]. Furthermore, Hiyama et al. identified specific Na_x_ sodium channels in the SFO that are important in sensing sodium and thus appear to be instrumental in regulating salt intake behaviour [55].

In summary, we have shown that drinking IS and HS solutions in SD rats cause a strong aversion to normally palatable saline solutions in two-bottle choice tests. We propose that this behaviour underlies the successful formation of CTA to salt solutions creating a new, exciting, and simple model to investigate salt appetite behaviours. The apparent destruction of the ancient evolutionary behaviour to naturally seek out and favourably ingest salty substances in our model is intriguing. Thus, increasing our understanding of the mechanisms responsible is of importance. We report here, lasting changes in mRNAs for markers of neuronal activity, peptide hormones and neuronal plasticity following RH though these effects were predominantly observed after both DHEx and HSEx. These data also imply that extreme care should be taken when planning experiments involving multiple episodes of HS exposure as the present findings clearly suggest that intake of HS solutions can alter salt appetite in SD rats.
